# Association of Genetic Risk for Schizophrenia With Nonparticipation Over Time in a Population-Based Cohort Study

**DOI:** 10.1093/aje/kww009

**Published:** 2016-05-10

**Authors:** Joanna Martin, Kate Tilling, Leon Hubbard, Evie Stergiakouli, Anita Thapar, George Davey Smith, Michael C. O'Donovan, Stanley Zammit

**Keywords:** Avon Longitudinal Study of Parents and Children, attrition bias, cohort studies, genetic risk, longitudinal studies, schizophrenia, study nonparticipation

## Abstract

Progress has recently been made in understanding the genetic basis of schizophrenia and other psychiatric disorders. Longitudinal studies are complicated by participant dropout, which could be related to the presence of psychiatric problems and associated genetic risk. We tested whether common genetic variants implicated in schizophrenia were associated with study nonparticipation among 7,867 children and 7,850 mothers from the Avon Longitudinal Study of Parents and Children (ALSPAC; 1991–2007), a longitudinal population cohort study. Higher polygenic risk scores for schizophrenia were consistently associated with noncompletion of questionnaires by study mothers and children and nonattendance at data collection throughout childhood and adolescence (ages 1–15 years). These associations persisted after adjustment for other potential correlates of nonparticipation. Results suggest that persons at higher genetic risk for schizophrenia are likely to be underrepresented in cohort studies, which will underestimate risk of this and related psychiatric, cognitive, and behavioral phenotypes in the population. Statistical power to detect associations with these phenotypes will be reduced, while analyses of schizophrenia-related phenotypes as outcomes may be biased by the nonrandom missingness of these phenotypes, even if multiple imputation is used. Similarly, in complete-case analyses, collider bias may affect associations between genetic risk and other factors associated with missingness.

Schizophrenia is a highly heritable and severely impairing neurodevelopmental disorder with onset typically in early adulthood. In a recent genome-wide association study (GWAS) meta-analysis of 34,241 schizophrenia cases, 45,604 controls, and 1,235 parent-offspring trios, the Schizophrenia Working Group of the Psychiatric Genomics Consortium reported 128 independent genome-wide significant single-nucleotide polymorphisms (SNPs) associated with risk of this disorder (1). Given that schizophrenia has a relatively low lifetime morbidity risk of about 0.7% in the general population (2), population-based cohort samples are unlikely to include many affected individuals. Such longitudinal studies may also be limited by nonparticipation at given time points, or even complete loss to follow-up.

There are multiple factors associated with nonparticipation in cohort studies, including socioeconomic adversity, male sex, and cognitive, emotional, and behavioral problems (3–6). Given that many of these factors are associated with risk of schizophrenia (7, 8), persons at higher risk for this disorder may also be more likely to be lost to follow-up, even before illness onset. Studies show that genetic risk of schizophrenia overlaps with risks of other psychiatric conditions, including bipolar disorder, major depressive disorder (MDD), attention-deficit/hyperactivity disorder, autism spectrum disorder, and intellectual disability (9–12). Given the breadth of phenotypic-genetic overlap, it is plausible that schizophrenia genetic risk predisposes people to a broad range of psychopathology that could be at subthreshold levels with regard to disorder diagnosis but result in higher levels of nonparticipation in longitudinal studies. Thus, schizophrenia could be related to nonparticipation in cohort studies either through clinical phenotypic features associated with the disorder or through genetic risk factors that affect an individual's premorbid state.

Missing data in longitudinal studies are associated with a loss of statistical power, but missing data may also cause bias in estimation. When an outcome affects participation, the assumption that outcome data are missing at random will not hold true. A given analysis is likely to be biased if the outcome variable is related to the probability of selection, conditional on the other variables included in the model (i.e., the outcome variable is missing not-at-random). Therefore, if increased risk of psychiatric, behavioral, or cognitive phenotypes is associated with nonparticipation and missing data in longitudinal studies, this could lead to biases in analyses which examine risk factors for psychiatric phenotypes. Bias due to missing data can also be looked upon as a form of collider bias (13, 14), where conditioning on “participation” or “being a complete case” can induce an association between any 2 factors that cause nonparticipation. So if increased genetic risk of a psychiatric phenotype causes nonparticipation, then complete-case analyses (i.e., restriction of analyses to samples with no missing data) could induce associations between genetic risk score and other risk factors for nonparticipation (e.g., sex, socioeconomic position).

To date, the issue of whether genetic risk of schizophrenia is directly associated with nonparticipation has not been examined in longitudinal data sets. However, it is an important question that needs to be addressed, especially in light of the recent increased interest in leveraging longitudinal population cohort data to investigate the genetic architecture of psychiatric and behavioral phenotypes (15). Our main aim in this study was to determine whether genetic risk from common genetic variants, as estimated by polygenic risk scores (PRS) for schizophrenia, based on the largest GWAS conducted to date (1), were associated with nonreturn of questionnaire data by children and parents and with nonattendance at clinic data collection assessments. We used data from a longitudinal population cohort study, the Avon Longitudinal Study of Parents and Children (ALSPAC). It was postulated that higher PRS in ALSPAC children and mothers would be associated with increased rates of nonparticipation. A secondary aim was to determine whether polygenic risk of schizophrenia was associated with nonparticipation above and beyond the associations of several known correlates of missing data: 1) family history of psychopathology (i.e., schizophrenia and MDD), 2) family socioeconomic factors, and 3) the index child's sex and behavioral and emotional problems.

## METHODS

ALSPAC is a large, well-characterized longitudinal data set (4, 16). ALSPAC originally recruited 14,541 pregnant women resident in Avon, United Kingdom, with expected delivery dates between April 1, 1991, and December 31, 1992. Among these pregnancies, there were 14,062 liveborn children, of whom 13,988 were alive at 1 year of age. An additional 713 children who would have been eligible but whose mothers did not enroll during pregnancy were enrolled after age 7 years, resulting in a total sample of 14,701 children alive at age 1 year. The study website contains details on all available data through a fully searchable data dictionary (17). Ethical approval for the study was obtained from the ALSPAC Ethics and Law Committee and local research ethics committees.

For the purposes of this study, only children who survived to age 1 year, had been enrolled in the study during the first phase of data collection, and had genotype data were included in the sample. This resulted in a sample of 7,867 children with imputed data for 2,543,887 SNPs after all quality control procedures were implemented. Imputation of genetic data was performed using HapMap release 22 in MACH 1.0.16 (18). Maternal genetic data were available for 7,850 mothers in the sample—a total of 2,543,887 SNPs after quality control. Details on quality control protocols for the genetic data are provided elsewhere (19). Genetic data for mother-child dyads were available for 5,262 mother-child pairs.

### Measures of participation

**Figure 1.**
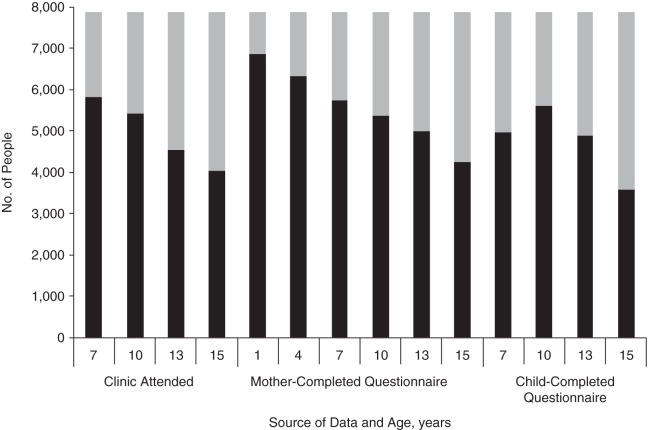
Data availability in a sample of mothers and children with genetic data from the Avon Longitudinal Study of Parents and Children, 1991–2007. Black indicates persons who participated in data collection at a given time point; gray indicates persons with missing data.

Yes/no variables were generated for whether each child had data available at each data collection time point (at the approximate ages of 7, 10, 13, and 15 years) from 3 sources: clinic attendance, mother-completed postal questionnaires, and child-completed postal questionnaires. Additionally, data on participation at 2 earlier time points for mother-completed questionnaires at ages 1 and 4 years (data available: yes/no) were used. These time points were selected to cover a reasonable spread of ages across childhood and adolescence for the study child, for the 3 types of data collection sources. The postal questionnaires contained questions covering a range of demographic, developmental, psychological, and health issues. Data collected at clinical assessments included cognitive and physiological measures (e.g., language abilities, blood pressure, lung function). Figure 1 shows the number of persons with available data for each time point. Additional information on how data were collected over time and how many people responded over time is available in the main study publications (4, 16).

### Potential correlates of nonparticipation

Family history, child-level, and socioeconomic variables previously shown to be associated with missing data were examined in relation to the above measures of participation. Binary variables for the presence of psychosis/schizophrenia (in first- or second-degree relatives) and maternal history of MDD were used as measures of family history of psychopathology. These were assessed using mother-completed questionnaires, which were completed during pregnancy with the index child. Child-level variables examined were sex and a total score for behavioral and emotional difficulties, as assessed by maternal report using the Strengths and Difficulties Questionnaire, at the approximate age of 4 years (20). The following socioeconomic variables were also examined: highest maternal educational level (Certificate of Secondary Education, vocational, O-levels, A-levels, or undergraduate degree), home ownership (yes/no), and parental socioeconomic position. Three socioeconomic categories were defined (low: unskilled workers/unemployed; medium: manual and nonmanual skilled/partially skilled workers; high: professional and managerial workers) on the basis of whichever reported parental occupation was higher-level; the occupations were classified using the United Kingdom Standard Occupational Classification (21).

### Discovery sample and calculating PRS

We used the largest schizophrenia GWAS published to date (1) to derive PRS in ALSPAC children and mothers. The *P* value threshold (*P*_T_) used to define risk alleles was *P* < 0.05, the threshold at which PRS maximally predict caseness in independent samples (9). Scores were calculated separately for ALSPAC children and mothers using imputed SNPs. First, SNPs in the discovery sample were filtered for a minor allele frequency greater than 1% and an INFO imputation score (a measure of the certainty and quality of the imputation results) greater than 0.9 (18). Linkage disequilibrium-based clumping was performed for SNPs overlapping discovery and ALSPAC samples in PLINK software, version 1.9 (22), using the following parameters: --clump-p1 0.5, --clump-r2 0.25, and --clump-kb 500. Additional quality control procedures were subsequently performed that checked for strand mismatches and substantial differences in allele frequencies (>0.1) between the Psychiatric Genomics Consortium schizophrenia sample (1) and the samples of ALSPAC children and mothers. Where appropriate, strand mismatches were corrected using the “flip” command in PLINK. Schizophrenia PRS were subsequently calculated for ALSPAC children and mothers using the --score function in PLINK. Scores were based on 35,768 SNPs in children and 35,756 SNPs in mothers. The scores were normally distributed and were standardized using *z*-score transformation to aid interpretation of the results.

### Statistical analysis

Analyses were performed in Stata, version 13.1 (StataCorp LP, College Station, Texas). Logistic regression analyses were used to estimate odds ratios and 95% confidence intervals for missingness per 1-standard-deviation increase in PRS. For each test, the Nagelkerke pseudo-*R*^2^ value is presented as an estimate of the amount of variance in outcome (missingness) explained by PRS. Analyses were performed separately for child PRS and maternal PRS for each of the 3 types of data collection (clinic attendance and child- and mother-completed questionnaires). Secondary analyses explored the addition of covariates, unique child and maternal associations, and whether the association with PRS changed over time. The latter analysis was conducted using generalized estimating equations, including an interaction term for the interaction of PRS with age, to examine change in the association over time.

## RESULTS

Table 1 displays descriptive statistics for covariates in the study sample, stratified by whether children had genetic data. Persons with genetic data were more likely to have a higher socioeconomic position, mothers with higher levels of education, parents who owned their own home, lower risk of maternal MDD, higher risk of a family history of psychosis, and lower scores on the Strengths and Difficulties Questionnaire than persons without genetic data.

**Table 1.** Distribution of the Study Sample According to Availability of Children's Genetic Data, Avon Longitudinal Study of Parents and Children, 1991–2007

Characteristic	Availability of Child's Genetic Data				Statistic^a^	
	Available		Not Available			
	No.	%	No.	%	χ^2^	*P* Value
Sex					0.20	0.66
Female	3,819	48.5	2,856	48.2		
Male	4,048	51.5	3,074	51.8		
Family socioeconomic status^b^					80.4	3.5*e*^−18^
Low	182	2.6	175	4.0		
Medium	4,809	68.7	3,232	74.3		
High	2,013	28.7	944	21.7		
Home ownership					313.3	4.1*e*^−70^
Renting/other	1,581	20.9	1,848	34.9		
Owned home	5,989	79.1	3,446	65.1		
Maternal education					298.1	2.7*e*^−63^
CSE	1,193	16.1	1,279	26.3		
Vocational	666	9.0	544	11.2		
O-levels	2,573	34.8	1,668	34.3		
A-levels	1,836	24.8	924	19.1		
Degree	1,134	15.3	442	9.1		
Family history of psychosis					53.4	2.7*e*^−13^
No	6,712	90.4	4,606	94.1		
Yes	715	9.6	291	5.9		
Maternal history of MDD					20.7	5.7*e*^−6^
No	6,819	92.0	4,376	89.6		
Yes	596	8.0	510	10.4		
SDQ total score^c,d^	8.8 (4.6)		9.2 (4.6)			

Abbreviations: CSE, Certificate of Secondary Education; MDD, major depressive disorder; SDQ, Strengths and Difficulties Questionnaire.

^a^ Descriptive statistics are based on 7,867 persons with genetic data available for analysis and 5,930 persons without genetic data in the analyses.

^b^ Low: unskilled worker/unemployed; medium: manual or nonmanual skilled/partially skilled worker; high: professional or managerial worker.

^c^ Values are presented as mean (standard deviation).

^d^ *t* = −3.8; *P* = 1.2*e*^−4^.

**Figure 2.**
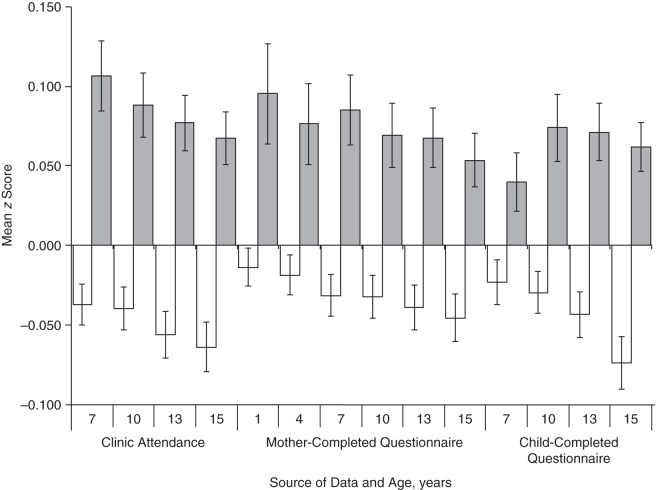
Polygenic risk scores (mean *z* score) for schizophrenia among children from the Avon Longitudinal Study of Parents and Children (1991–2007), depending on data availability. White bars represent persons with data available; gray bars represent persons with missing data. Error bars show standard errors.

**Figure 3.**
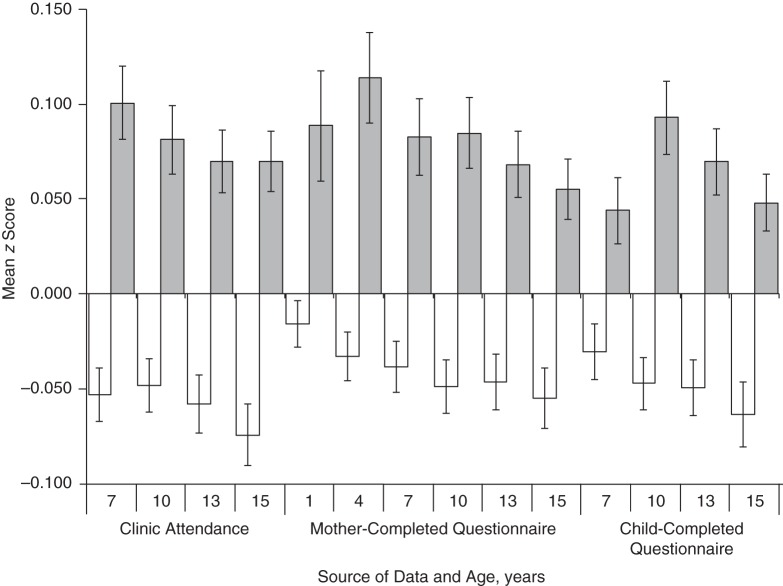
Polygenic risk scores (mean *z* score) for schizophrenia among mothers from the Avon Longitudinal Study of Parents and Children (1991–2007), depending on data availability. White bars represent persons with data available; gray bars represent persons with missing data. Error bars show standard errors.

There was strong evidence that higher schizophrenia PRS in the children were associated with missing data for all measures and time points (see Table 2). The odds ratios reflected an increased likelihood of missing data per 1-standard-deviation increase in schizophrenia PRS. PRS in mothers were similarly associated with missing data for all time points (see Table 2). The mean differences in PRS for persons with and without missing data are shown in Figures 2 and 3 for PRS in children and mothers, respectively.

**Table 2.** Association of Schizophrenia Polygenic Risk Scores With Missing Data Among Children and Mothers From the Avon Longitudinal Study of Parents and Children, 1991–2007

Source of Data and Age, years	Child PRS (*n* = 7,867)				Maternal PRS (*n* = 7,850)			
	OR	95% CI	*P* Value	Pseudo-*R*^2^	OR	95% CI	*P* Value	Pseudo-*R*^2^
Clinic attendance								
7	1.16	1.10, 1.22	2.0*e*^−8^	0.0035	1.17	1.11, 1.22	1.0*e*^−8^	0.0042
10	1.14	1.08, 1.19	1.5*e*^−7^	0.0028	1.14	1.09, 1.19	3.0*e*^−8^	0.0030
13	1.14	1.09, 1.20	1.0*e*^−8^	0.0032	1.14	1.09, 1.19	2.0*e*^−8^	0.0030
15	1.14	1.09, 1.19	1.0*e*^−8^	0.0031	1.16	1.11, 1.21	1.0*e*^−8^	0.0038
Mother-completed questionnaire								
1	1.12	1.04, 1.19	1.3*e*^−3^	0.0017	1.11	1.04, 1.18	9.3*e*^−4^	0.0016
4	1.10	1.04, 1.16	7.8*e*^−4^	0.0014	1.16	1.10, 1.22	5.0*e*^−8^	0.0036
7	1.12	1.07, 1.18	4.3*e*^−6^	0.0023	1.13	1.08, 1.18	5.7*e*^−7^	0.0026
10	1.11	1.06, 1.16	2.8*e*^−5^	0.0018	1.14	1.09, 1.20	1.0*e*^−8^	0.0032
13	1.11	1.06, 1.17	5.1*e*^−6^	0.0020	1.12	1.07, 1.17	5.8*e*^−7^	0.0024
15	1.10	1.06, 1.15	1.2*e*^−5^	0.0018	1.12	1.07, 1.17	1.1*e*^−6^	0.0022
Child-completed questionnaire								
7	1.07	1.02, 1.12	7.0*e*^−3^	0.0007	1.08	1.03, 1.13	1.1*e*^−3^	0.0010
10	1.11	1.06, 1.17	3.1*e*^−5^	0.0018	1.15	1.10, 1.21	1.0*e*^−8^	0.0034
13	1.12	1.07, 1.17	7.9*e*^−7^	0.0023	1.13	1.08, 1.18	2.1*e*^−7^	0.0025
15	1.15	1.10, 1.20	1.0*e*^−8^	0.0033	1.12	1.07, 1.17	1.0*e*^−6^	0.0022

Abbreviations: CI, confidence interval; OR, odds ratio; PRS, polygenic risk scores.

**Figure 4.**
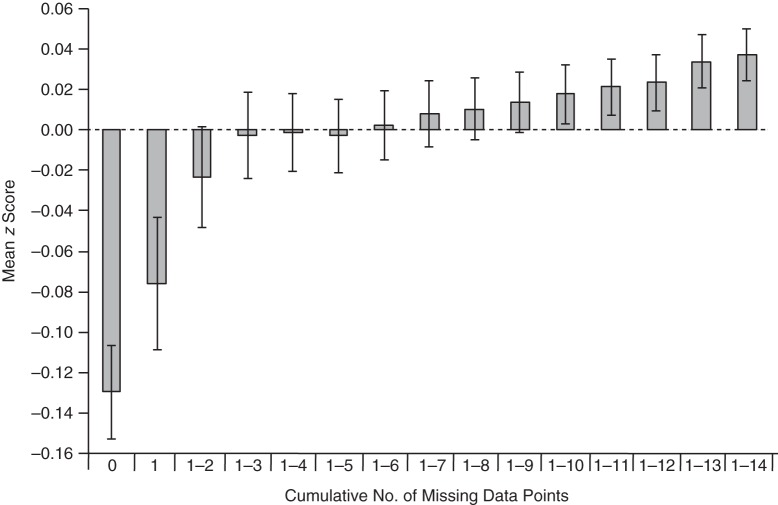
Polygenic risk scores (mean *z* score) for schizophrenia among children from the Avon Longitudinal Study of Parents and Children (1991–2007), according to the number of missing data points. The *x*-axis displays categories of persons with no missing data, missing data for only 1 time point, missing data for 1–2 time points, missing data for 1–3 time points, and so on. Error bars show standard errors.

**Figure 5.**
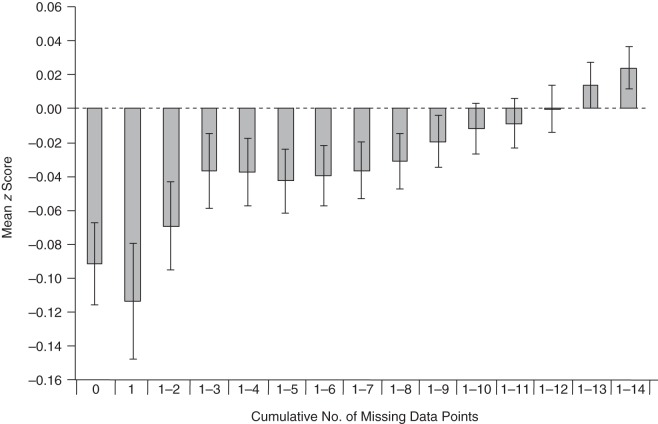
Polygenic risk scores (mean *z* score) for schizophrenia among mothers from the Avon Longitudinal Study of Parents and Children (1991–2007), according to the number of missing data points. The *x*-axis displays categories of persons with no missing data, missing data for only 1 time point, missing data for 1–2 time points, missing data for 1–3 time points, and so on. Error bars show standard errors.

Figures 4 and 5 display mean PRS for schizophrenia in children and mothers, depending on the cumulative number of missing data points. A linear regression of the number of missing data points on schizophrenia PRS showed that PRS were associated with an increase of 0.07 missing data points (0.08 in mothers) per 1-standard-deviation increase in PRS.

Results of multivariable regression analyses of the association between schizophrenia PRS and missing data, after adjustment for family history of MDD or schizophrenia, child variables (sex and total Strengths and Difficulties Questionnaire problems), and socioeconomic variables (family socioeconomic position, maternal education, and home ownership), can be found in Table 3. Complete data on all variables were available for 5,601 children and 5,347 mothers. PRS for schizophrenia in children continued to show an association with availability of data for the majority of data collection time points (except mother-completed questionnaires at age 4 years and child-completed questionnaires at age 7 years), even after accounting for the known correlates of missing data in longitudinal research. Similar results were found for PRS calculated in mothers (see Table 3), with strong evidence of association between maternal PRS and data availability at the majority of data collection time points, after accounting for the known correlates of nonparticipation. On the whole, effect sizes for the association of PRS with availability of data at the collection time points were not changed upon the addition of these correlates to the models, although the evidence for association was marginally stronger than in the unadjusted models. (See Web Table 1, available at http://aje.oxfordjournals.org/, for results of unadjusted analyses confined to persons with no missing data.)

**Table 3.** Association of Schizophrenia Polygenic Risk Scores With Missing Data Among Children and Mothers, After Adjustment for Known Correlates of Study Nonparticipation,^a^ Avon Longitudinal Study of Parents and Children, 1991–2007

Source of Data and Age, years	Child PRS (*n* = 5,601)				Maternal PRS (*n* = 5,347)			
	OR	95% CI	*P* Value	Pseudo-*R*^2^	OR	95% CI	*P* Value	Pseudo-*R*^2^
Clinic attendance								
7	1.15	1.07, 1.24	1.9*e*^−4^	0.0029	1.18	1.10, 1.26	1.4*e*^−6^	0.0043
10	1.13	1.06, 1.20	3.8*e*^−4^	0.0022	1.13	1.06, 1.20	2.8*e*^−4^	0.0023
13	1.15	1.08, 1.21	3.6*e*^−6^	0.0031	1.09	1.03, 1.16	2.7*e*^−3^	0.0014
15	1.15	1.09, 1.22	7.7*e*^−7^	0.0032	1.13	1.06, 1.19	4.2*e*^−5^	0.0023
Mother-completed questionnaire								
1	1.24	1.06, 1.44	6.2*e*^−3^	0.0048	1.10	0.96, 1.27	0.17	0.0011
4	1.01	0.90, 1.14	0.88	0.0000	1.27	1.12, 1.44	1.5*e*^−4^	0.0068
7	1.11	1.03, 1.20	9.0*e*^−3^	0.0016	1.10	1.02, 1.19	0.013	0.0014
10	1.09	1.02, 1.17	0.013	0.0012	1.13	1.05, 1.20	6.5*e*^−4^	0.0022
13	1.08	1.02, 1.15	0.013	0.0010	1.10	1.04, 1.17	2.3*e*^−3^	0.0015
15	1.11	1.05, 1.18	4.5*e*^−4^	0.0018	1.11	1.05, 1.18	5.5*e*^−4^	0.0017
Child-completed questionnaire								
7	1.04	0.97, 1.10	0.25	0.0002	1.03	0.97, 1.10	0.30	0.0002
10	1.10	1.03, 1.19	6.5*e*^−3^	0.0015	1.18	1.10, 1.26	4.9*e*^−6^	0.0040
13	1.11	1.04, 1.18	9.4*e*^−4^	0.0017	1.12	1.05, 1.19	3.7*e*^−4^	0.0021
15	1.15	1.09, 1.22	6.0*e*^−7^	0.0032	1.10	1.04, 1.17	7.0*e*^−4^	0.0015

Abbreviations: CI, confidence interval; OR, odds ratio; PRS, polygenic risk scores.

^a^ Family history of schizophrenia or depression, child sex and behavioral/emotional problems, family socioeconomic position, maternal educational level, and home ownership.

### Secondary analyses

In order to further examine the source of the genetic association with nonparticipation, we repeated the analyses while including both child and maternal PRS in the same model, for child-mother pairs with available genetic data (*n* = 5,262). The correlation between children's and mothers' scores was 0.509. As can be seen from Table 4, children's PRS were associated with missing data independently of the association of maternal PRS for the majority of data collection time points (except for mother-completed questionnaires at age 4 years). There was no evidence of association of maternal PRS with nonparticipation, above and beyond the variance shared with child PRS. On the whole, odds ratios were higher for child PRS than for maternal PRS.

**Table 4.** Association of Schizophrenia Polygenic Risk Scores With Missing Data Among Children and Mothers, After Adjustment for Shared Associations, Avon Longitudinal Study of Parents and Children, 1991–2007

Source of Data and Age, years	Child PRS (*n* = 5,262)				Maternal PRS (*n* = 5,262)			
	OR	95% CI	*P* Value	Pseudo-*R*^2^	OR	95% CI	*P* Value	Pseudo-*R*^2^
Clinic attendance								
7	1.12	1.04, 1.21	3.6*e*^−3^	0.0016	1.07	1.00, 1.16	0.066	0.0006
10	1.14	1.06, 1.23	5.3*e*^−4^	0.0021	1.03	0.96, 1.11	0.43	0.0001
13	1.15	1.08, 1.23	4.0*e*^−5^	0.0025	1.02	0.95, 1.09	0.62	<0.0001
15	1.11	1.04, 1.19	1.1*e*^−3^	0.0015	1.06	1.00, 1.13	0.056	0.0005
Mother-completed questionnaire								
1	1.12	1.01, 1.24	0.033	0.0013	1.05	0.95, 1.16	0.36	0.0003
4	1.07	0.98, 1.17	0.11	0.0006	1.04	0.96, 1.13	0.33	0.0002
7	1.16	1.07, 1.25	1.5*e*^−4^	0.0026	0.97	0.90, 1.04	0.35	0.0002
10	1.13	1.05, 1.21	9.3*e*^−4^	0.0018	1.00	0.93, 1.07	0.97	<0.0001
13	1.09	1.02, 1.16	0.016	0.0009	1.04	0.97, 1.11	0.26	0.0002
15	1.10	1.04, 1.18	2.5*e*^−3^	0.0013	1.00	0.94, 1.07	0.91	<0.0001
Child-completed questionnaire								
7	1.07	1.00, 1.14	0.048	0.0006	0.97	0.91, 1.04	0.34	0.0001
10	1.11	1.03, 1.20	4.9*e*^−3^	0.0013	1.05	0.98, 1.13	0.19	0.0003
13	1.13	1.05, 1.21	4.9*e*^−4^	0.0018	1.03	0.96, 1.10	0.38	0.0001
15	1.17	1.09, 1.24	2.1*e*^−6^	0.0031	1.02	0.96, 1.09	0.55	0.0001

Abbreviations: CI, confidence interval; OR, odds ratio; PRS, polygenic risk scores.

Finally, we estimated the population-averaged association between schizophrenia PRS and missing data across time points and examined whether this changed over time (see Web Table 2). The results suggested that the association of child PRS with child-completed questionnaires increased over time, but there was no evidence that the association with other data sources changed over time.

## DISCUSSION

The results of this study show a consistent pattern of association between increased PRS for schizophrenia in children and mothers from the ALSPAC general population sample and nonparticipation in data collection at time points across ages 1–15 years. The magnitudes of these associations remained similar after adjustment for several known correlates of nonparticipation (i.e., family history of schizophrenia or depression, child sex and behavioral/emotional problems, family socioeconomic position, maternal educational level, and home ownership (3–6)). Together, these results suggest that analyses of schizophrenia (and genetically related psychiatric phenotypes) as outcomes in longitudinal population cohort studies are likely to be underpowered, given that persons with a genetic predisposition to these phenotypes will be underrepresented in longitudinal population cohorts. They also indicate that some analyses of schizophrenia and related outcomes may be biased because data for these outcomes may be missing not-at-random.

Given the low prevalence of schizophrenia in the population (2) and the high pleiotropy of genetic risk of schizophrenia (10–12), the results further imply that nonparticipation in longitudinal research may be associated with other psychiatric disorders (e.g., MDD or attention-deficit/hyperactivity disorder), as well as subclinical psychopathology or related phenotypes (e.g., personality traits, cognitive function), even in the absence of a psychiatric diagnosis. The observed association between genetic risk of schizophrenia and study nonparticipation is likely to be the result of an influence of schizophrenia risk alleles on phenotypes beyond those generally considered directly related to the disorder (e.g., other psychopathology or behavioral, cognitive, or personality phenotypes), since the association was seen by the age of 7 years—an age at which the probability of children in this population sample having schizophrenia or schizophrenia-spectrum disorders was very low. This is an important observation that is likely to be relevant for analyses of any population data, particularly in longitudinal studies. Given the high levels of pleiotropy for psychiatric and behavioral phenotypes, it is highly plausible that the mechanism for genetic risk of schizophrenia operates on nonparticipation through such associated phenotypes, which are collectively more common in the population than schizophrenia alone. Additional research is needed to further investigate the specific mechanisms through which this genetic risk is associated with nonparticipation in population studies. Another possibility includes active gene-environment correlation, whereby genetic risk influences likelihood of exposure to environmental factors.

Regardless of the exact mechanisms, the plausible importance of subclinical phenotypes in nonparticipation further supports a quantitative trait model of psychiatric phenotypes, suggesting that there is no clear boundary between the presence and absence of an adverse phenotypic outcome and that genetic risk is probably distributed along a continuous liability scale. Indeed, a recent study of autism and variation in related social-communicative traits in the population supported such a model, by highlighting that both common and rare genetic variants operate on a continuum of behavioral and developmental traits (23).

If genetic risk of schizophrenia is associated with study nonparticipation but schizophrenia phenotype is not (conditional on genetic risk), then analyses of schizophrenia as an outcome in longitudinal studies would probably not be biased if genetic risk were included as an exposure in the analytical model. If, on the other hand, a phenotype is associated with nonparticipation independently of genetic risk, then it is likely that analyses of schizophrenia as an outcome will be biased by the nonrandom missingness of this phenotype even with inclusion of genetic risk in the model. Furthermore, given that schizophrenia genetic risk is associated with attrition in cohort studies, complete-case analyses could lead to collider bias, affecting associations between genetic risk score and other exposures, such as substance use, that also lead to nonparticipation.

It is clear that further work is needed to elucidate the mechanism through which genetic risk operates to result in a given phenotype, such as nonparticipation in a longitudinal study. The method of PRS analysis provides the potential to help with this process. For instance, the association between increased genetic risk of schizophrenia and schizophrenia as an outcome is partly mediated through family history of psychiatric illness (24), and as GWAS sample sizes continue to increase, such analyses are likely to shed further light on our understanding of disease risk.

In the secondary analyses including both child and maternal PRS in a single analytical model, maternal PRS appeared not to contribute unique variance to the association with missing data. However, there were consistent associations of child PRS with nonparticipation at all data collection time points. This finding suggests that additional genetic risk factors beyond those carried by the mother influence the likelihood of mothers and children not returning questionnaires or attending the clinic visits.

It is possible that the current study underestimated the effect size of the association between PRS in mothers and nonparticipation because some mothers with higher PRS may have not enrolled in the study at all or may not have provided a DNA sample and so were not part of the analyses. Genetic data were available for 57% of the sample for participants alive at 1 year, and these individuals differed with regard to all covariates except sex in comparison with those without genetic data (see Table 1). DNA samples were collected from ALSPAC children and mothers over a number of years and came from a variety of sources, including umbilical cord blood (at birth), whole blood drawn when the child was 7 years of age, and cell lines generated from blood collected at ages 9–17 years. Thus, it is likely that persons at higher genetic risk for schizophrenia could have dropped out of data collection prior to a DNA sample being obtained. This is an important limitation of the results and is likely to have resulted in underestimation of the effect sizes. Indeed, there was a clear pattern of persons with missing genetic data being more likely to have missing phenotype data across all of the data collection time points examined (see Web Table 3).

There were other limitations as well. Firstly, genetic data from fathers were not available. Secondly, as with other studies utilizing the PRS method, the effect sizes (particularly in terms of variance explained) were not very high. A third issue is that other potential correlates of nonparticipation (e.g., child's intelligence quotient (IQ)) were not taken into account in this study. The reason for this is that we made an effort to maximize the number of samples that could be analyzed by utilizing correlates of nonparticipation gathered early on in the study. IQ was measured when the children were 7 years old; therefore, including this variable drastically reduces the sample size and power of the analyses. However, when IQ is included, the effect sizes remain comparable to those shown in Table 3 (results available from first author on request).

Despite these limitations, the results of this study show a consistent pattern of association between genetic risk of schizophrenia and nonparticipation by study mothers and children in data collection at numerous time points across childhood and adolescence. These results have important implications for genetic studies that utilize psychiatric-related phenotypes ascertained using a longitudinal cohort design and possibly also for studies conducted in the general population, which may suffer from selection biases. The results suggest that persons at higher genetic risk for schizophrenia and related phenotypes are likely to be underrepresented in longitudinal studies, which may decrease the statistical power for any analyses of schizophrenia and other related phenotypes, both in terms of categorical diagnoses and in terms of quantitative population traits. Our results suggest that analyses with schizophrenia as an outcome may be biased by the nonrandom missingness of this phenotype, especially if genetic risk factors are not taken into account. It is possible that the missing-at-random assumption of multiple imputation models may still not be met even after inclusion of variables related to family history, socioeconomic position, and childhood behavioral and emotional problems. These results open up possibilities for future research into nonparticipation in longitudinal studies—for example, regarding whether inclusion of genetic risk scores in imputation of missing data might allow associations to be estimated with less bias.

## Supplementary Material

Web Material
