# Supplementary material for: Association of Genetic Risk for Schizophrenia With Nonparticipation Over Time in a Population-Based Cohort Study
Source: Am J Epidemiol. 2016 May 10;183(12):1149–58. doi: 10.1093/aje/kww009 (PMC4908211; doi:10.1093/aje/kww009)
Supplement: Web Material [file supp_183_12_1149__index.html]

Association of Genetic Risk for Schizophrenia With Nonparticipation Over Time in a Population-Based Cohort Study — Web Material 

# Association of Genetic Risk for Schizophrenia With Nonparticipation Over Time in a Population-Based Cohort Study

## Web Material

Web Material

- Web Material - Pdf file
