# Supplementary material for: Association of Genetic Risk for Schizophrenia With Nonparticipation Over Time in a Population-Based Cohort Study
Source: Am J Epidemiol. 2016 May 10;183(12):1149–58. doi: 10.1093/aje/kww009 (PMC4908211; doi:10.1093/aje/kww009)
Supplement: Web Material [file supp_kww009_kww009supp.pdf]

WEB MATERIAL

Web Table 1. Association of schizophrenia polygenic risk scores (in children and mothers) with missing data; analysis limited to individuals with no missing covariate data

Source of Data	Age, years	Child Polygenic Risk Scores (*n* = 5,601)				Maternal Polygenic Risk Scores (*n* = 5,347)			
		OR	95% CI	*P*	Pseudo-R^2	OR	95% CI	*P*	Pseudo-R^2
Clinic attendance	7	1.13	1.05-1.22	7.7E-4	0.0024	1.17	1.09-1.25	4.0E-6	0.0039
	10	1.11	1.04-1.19	1.2E-3	0.0018	1.12	1.05-1.19	4.2E-4	0.0021
	13	1.13	1.07-1.19	2.5E-5	0.0025	1.08	1.02-1.15	5.6E-3	0.0011
	15	1.13	1.07-1.19	1.2E-5	0.0026	1.12	1.06-1.18	9.2E-5	0.0021
Mother-completed questionnaire	1	1.22	1.05-1.41	0.010	0.0042	1.09	0.95-1.25	0.22	0.0009
	4	1.00	0.88-1.12	0.94	<0.0001	1.25	1.10-1.41	4.6E-4	0.0058
	7	1.09	1.01-1.18	0.026	0.0011	1.09	1.01-1.18	0.020	0.0012
	10	1.07	1.00-1.14	0.047	0.0007	1.11	1.04-1.19	1.8E-3	0.0018
	13	1.06	1.00-1.13	0.052	0.0006	1.09	1.02-1.15	8.6E-3	0.0011
	15	1.08	1.02-1.14	5.4E-3	0.0011	1.09	1.03-1.15	2.9E-3	0.0013
Child-completed questionnaire	7	1.03	0.96-1.09	0.42	0.0001	1.03	0.96-1.09	0.42	0.0001
	10	1.09	1.02-1.17	0.017	0.0011	1.16	1.09-1.24	1.4E-5	0.0036
	13	1.09	1.03-1.16	4.2E-3	0.0013	1.10	1.04-1.17	1.2E-3	0.0017
	15	1.12	1.06-1.18	2.6E-5	0.0023	1.08	1.02-1.14	5.1E-3	0.0011

Abbreviations: CI, confidence interval; OR, odds ratio.

Web Table 2. Association of schizophrenia polygenic risk scores (in children and mothers) with missing data across time, using generalized estimating equations

Source of Data	Variable	Child Polygenic Risk Scores (*n* = 7,867)			Maternal Polygenic Risk Scores (*n* = 7,850)		
		**OR**	**95% CI**	***P***	**OR**	**95% CI**	***P***
Clinic attendance	PRS	1.16	1.06-1.27	8.6E-04	1.17	1.08-1.27	7.1E-05
	Age	1.14	1.13-1.14	1.0E-08	1.09	1.09-1.10	1.0E-08
	PRS × age	1.00	0.99-1.00	0.69	1.00	0.99-1.00	0.56
Mother-completed questionnaire	PRS	1.11	1.05-1.18	2.6E-04	1.14	1.08-1.20	1.5E-06
	Age	1.12	1.11-1.12	1.0E-08	1.12	1.11-1.12	1.0E-08
	PRS × age	1.00	0.99-1.00	0.95	1.00	0.99-1.00	0.74
Child-completed questionnaire	PRS	1.02	0.94-1.10	0.71	1.09	1.00-1.18	0.034
	Age	1.10	1.09-1.10	1.0E-08	1.08	1.08-1.09	1.0E-08
	PRS × age	1.01	1.00-1.01	0.016	1.00	0.99-1.00	0.44

Abbreviations: CI, confidence interval; OR, odds ratio; PRS, polygenic risk scores.

Web Table 3. Number of individuals not participating at each data collection time point, depending on availability of child's genetic data

Source of Data	Age, years	Genetic Data Available *N* (%)	Genetic Data Not Available *N* (%)	χ^2 *
Clinic attendance	7	2039 (25.9%)	4103 (67.2%)	2400
	10	2455 (31.2%)	4353 (71.3%)	2200
	13	3325 (42.3%)	4813 (78.8%)	1900
	15	3837 (48.8%)	4892 (80.1%)	1400
Mother-completed questionnaire	1	1001 (12.7%)	1918 (31.4%)	726
	4	1555 (19.8%)	2706 (44.3%)	976
	7	2133 (27.1%)	3611 (59.1%)	1500
	10	2508 (31.9%)	3960 (64.8%)	1500
	13	2888 (36.7%)	4242 (69.4%)	1500
	15	3627 (46.1%)	4627 (75.7%)	1200
Child-completed questionnaire	7	2913 (37.0%)	3966 (64.9%)	1100
	10	2260 (28.7%)	3791 (62.1%)	1600
	13	2989 (38.0%)	4172 (68.3%)	1300
	15	4287 (54.5%)	4788 (78.4%)	861

* All *P*’s < 0.001.
